# Mother and adolescent expressed emotion and adolescent internalizing and externalizing symptom development: a six-year longitudinal study

**DOI:** 10.1007/s00787-015-0772-7

**Published:** 2015-09-29

**Authors:** William W. Hale, Elisabetta Crocetti, Stefanie A. Nelemans, Susan J. T. Branje, Pol A. C. van Lier, Hans M. Koot, Wim H. J. Meeus

**Affiliations:** Research Centre Adolescent Development, Utrecht University, P.O. Box 80140, 3508 TC Utrecht, The Netherlands; Faculty of Psychology and Education, VU University Amsterdam, Van der Boechorstraat 1, 1081 BT Amsterdam, The Netherlands; Department of Developmental Psychology, Tilburg University, P.O. Box 90153, 5000 LE Tilburg, The Netherlands

**Keywords:** Adolescent, Expressed emotion, Externalizing symptoms, Internalizing symptoms, Longitudinal, Mother

## Abstract

In expressed emotion (EE) theory, it is held that high EE household environments enhance adolescent psychopathological distress. However, no longitudinal study has been conducted to examine if either the mother’s EE or the adolescent’s perception of EE predicts adolescent internalizing and externalizing symptom dimensions (an EE effect model) or vice versa (psychopathological effect model) together in one model. To unravel the reciprocal influences of maternal and adolescent perceived EE to adolescent internalizing and externalizing symptom dimensions, we tested two (i.e., one for internalizing and one for externalizing) cross-lagged panel models. In this study, it was found that both internalizing and externalizing symptom dimensions predicted the adolescent’s perception of maternal EE as well as the mother’s own rated EE criticism over time. The findings of this study should give both researchers and therapists a reason to reevaluate only using the EE effects model assumption in future EE studies.

## Introduction

In the study of the effects environmental factors have on the development of adolescent psychopathology, one line of research that has received prominent attention has been the study of expressed emotion. Expressed emotion (or: EE) is literally a measure of the emotions expressed by a significant family member or spouse to an individual suffering from a psychopathological disorder and is used as a predictor of relapse following hospitalization [1]. This line of research, started by George Brown in the 1950s, was first used to better understand why certain persons relapsed back into schizophrenia when living with a significant relative or spouse after hospitalization and why others did not relapse (e.g., Brown, 1985) [1]. It was found that certain aspects of the emotional climate at home seemingly augment the patient’s symptoms, leading to relapse.

Initially, EE data were collected by interviewing the important family member with the Camberwell Family Interview (CFI) [2] as to his/her interaction with the patient. Scoring of the CFI EE interview focuses on three domains in which negative interpersonal interactions can occur in the household environment between the mother and the adolescent. Specifically, these domains are the mother’s emotional over-involvement, hostility, and criticism to the adolescent. In previous cross-sectional studies based on the CFI interview of EE, high maternal EE has been found to be associated with high levels of adolescent internalizing [3, 4] and externalizing symptoms [5, 6]. This has led EE researchers to take the stance that mothers that express a great amount of EE on any or all of these three domains (which is commonly referred to in the literature as high EE) enhances an adolescent’s symptom development; an EE effects model [7]. More importantly, many of the present-day EE therapeutic interventions are based on the assumption that maternal EE enhances adolescent symptom development [2]. However, studies on the direction of effects between psychopathology and EE are not necessarily consistent, and therefore the question arises whether the parental EE effects model is correct.

Indeed, a number of recent adolescent–parent EE studies have explicitly challenged the assumed direction of effects [8–10]. These studies have found both bidirectional and child effects models in addition to maternal effects models as well. While earlier EE studies only interviewed of the provider of the EE, these recent adolescent–mother EE studies have used an EE questionnaire as opposed to an interview. Hence both the recipient and provider of the EE provided answers about the family EE climate. The findings of these recent EE studies have challenged the findings of previous EE studies that only examined unidirectional maternal effects. This use of EE questionnaires, which are quickly administrated and scored, as opposed to EE interviews (e.g., the CFI interview takes approximately 2 h to conduct and approximately 3 h to code), has allowed for prospective, longitudinal studies addressing effects of EE (as opposed to the traditional retrospective, cross-sectional studies of EE interviews based on the CFI).

One of the most used EE questionnaires that have been employed in recent adolescent–mother EE studies is the level of expressed emotion questionnaire (LEE) [11–13]. This questionnaire, much like the original CFI EE interview, focuses on the perspective of the person being asked about the family EE climate. Specifically, the 38-item version of the LEE questionnaire comprised four EE dimension scales. These four scales are criticism, which is related to the CFI EE domain of criticism, intrusiveness, which is related to the CFI EE domain of over-involvement, irritation, which is related to the CFI EE domain of hostility, and lack of emotional support, which purports to measure a general emotional negativity common in the EE household environment. Importantly, while in the original EE CFI interview studies it was the mother who was interviewed about the EE household environment, now both the mother and the adolescent can be asked with the LEE questionnaire.

However, most recent longitudinal EE studies have focused on just one person’s perspective, either that of the mother or that of the adolescent. For example, a longitudinal study by Hale et al. [14] of the mothers’ EE suggests that it is the course of the internalizing and externalizing symptoms of adolescents from the general community that affects maternal EE, and not the mothers’ perceived EE influencing the course of the adolescents’ symptoms. This study of Hale et al. [14] did not include the adolescent’s perceived EE.

An important first step toward a longitudinal study that did include both the mother’s and the adolescent’s perspectives of the mother’s EE, in one and the same study, found a bidirectional effect between adolescent depression and generalized anxiety disorder symptom dimensions and perceived maternal EE criticism [10]. This study also found stronger child effects (that of the adolescent internalizing symptom dimensions predicting perceived maternal EE criticism) than maternal effects. However, this study only focused on perceived maternal EE criticism and did not include either the other perceived EE factors (such as lack of emotional support, intrusiveness, and irritation) or adolescent externalizing symptom dimensions. So while this aforementioned study is an important first step, in order to understand if high EE household environments help create or enhance psychopathological symptomatology in adolescents (as is contented by previous EE CFI interview studies) or if the reverse is the case, measures of both adolescent and mother perceived EE need to be included in a longitudinal model addressing adolescent internalizing and externalizing symptom dimensions.

To tackle this omission in the EE literature, the goal of the present 6-year, longitudinal study is to include both the parent’s and the adolescent’s perspectives of all the parent’s EE components in order to disentangle the effects various parental EE components have on the adolescent’s internalizing and externalizing symptom development and vice versa. In order to accomplish this goal, data were used from the ongoing, longitudinal study of Research on Adolescent Development and Relationships (or: RADAR). The RADAR study collected LEE data from both adolescents and their mothers. While the longitudinal LEE study of mothers by Hale et al. [14] used this same database after 3 years of data collection, the present 6-year longitudinal study now also includes both the adolescent and mother responses on all LEE scales after 6 years of data collection so that in one and the same statistical model the effects of EE of both respondents can be compared to one another. By including the responses of both the receiver of EE (the adolescent) and the provider of EE (the mother) in the same model, the relative effects of EE on adolescent’s internalizing and externalizing symptom development can be better understood.

## Methods

### Participants

Data were drawn from an ongoing longitudinal study conducted in the Netherlands and entitled RADAR (Research on Adolescent Development and Relationships) that collected data on adolescents and their mothers. For the current study, we used six waves of annual questionnaire data that were collected among 497 Dutch (283 boys and 214 girls) adolescents and their mothers. At the first measurement wave, adolescents were in the first year of junior high school and were 13.03 years old, on average (SD = 0.46). Mothers were 44.41 years, on average (SD = 4.45). Since this study followed the adolescents for 6 years, most of their adolescent development was followed from the age of 13 to 18 years.

Participants provided information for six waves, with 1-year intervals between each wave. Of the original sample, 425 families (86 %) were still involved in the study at Wave 6, and the average participation rate over the six waves was 90 %. More specifically, the average percentage of missing cases was 11.26, 12.34, and 11.21 % for maternal LEE, adolescent LEE, and adolescent problem behavior questionnaires, respectively. For each set of these variables, Little’s Missing Completely at Random (MCAR) [15] test produced a statistically non-significant value (maternal LEE: *χ*^2^/*df* = 0.92, *p* = 0.810; adolescent LEE: *χ*^2^/*df* = 1.08, *p* = 0.118; adolescent problem behaviors: *χ*^2^/*df* = 1.08, *p* = 0.221), suggesting that data were missing completely at random. Therefore, all 497 adolescent–mother dyads were included in the analyses and missing data were estimated in Mplus 7.11 [16] using the Full Information Maximum Likelihood method [17].

### Procedure

Before the start of the study, adolescents and their mothers received written information about the research and they provided written informed consent. Each year, the adolescents and their mothers filled in questionnaires during home-visits. Trained research assistants provided verbal instructions, given just prior to the filling in of the questionnaires to compliment the written instructions printed above each questionnaire. Other research assistants conducted the data entry to ensure that the data remained anonymous. This study was approved by the medical ethics committee of University Medical Center Utrecht (The Netherlands).

### Measures

#### Adolescent and maternal expressed emotion

We employed the 38-item Dutch version of the level of expressed emotion, which takes approximately 5 min to complete [14]. The LEE assesses four EE dimensions: lack of emotional support (19 items), intrusiveness (7 items), irritation (7 items), and criticism (5 items) [14]. The questionnaire, filled in by both the adolescent and the mother, is scored on a four-point scale ranging from 1 = ‘untrue’ to 4 = ‘true,’ so high LEE scores correspond to perceived high levels of lack of emotional support, intrusiveness, irritation, and criticism of the parents (for the adolescent’s version of the LEE) or the mother’s perception of her own high expressed emotion (for the mother’s version of the LEE). The questionnaire items for the mother and adolescent versions refer to the same content but are worded somewhat differently to reflect receiving EE from his/her parents on the part of the adolescent or providing EE on the part of the mother. Sample items from the adolescent and mother versions of the LEE (respectively) include the following: “Accuse me of exaggerating when I say I’m unwell/Accuse my child of exaggerating when he/she says he/she is unwell” (for lack of emotional support), “Are always nosing into my business/Am always nosing into my child’s business” (for intrusiveness), “Fly off the handle when I don’t do something well/Fly off the handle when my child doesn’t do something well” (for irritation), and “Are critical of me/Am critical of my child” (for criticism),

With respect to the psychometric qualities of the LEE, prior studies demonstrated with confirmatory factor analyses that the factor structure of the LEE applied well to both adolescents [18] and mothers [14].

In this study, the range of Cronbach’s internal consistency coefficients of the LEE subscales across the six waves were as follows: lack of emotional support (adolescent: *α*_s_ = 0.84–0.93, mother: *α*_s_ = 0.78–0.83); intrusiveness (adolescent: *α*_s_ = 0.66–0.86, mother: *α*_s_ = 0.82–0.86); irritation (adolescent: *α*_s_ = 0.77–0.87, mother: *α*_s_ = 0.76–0.80); and criticism (adolescent: *α*_s_ = 0.73–0.81, mother: *α*_s_ = 0.57–0.59).

Both the mother and the adolescent versions of the LEE (and the scale-scoring key) are available by request from the first author.

#### Adolescent internalizing symptoms

Adolescent internalizing symptom dimensions were measured with the 23-item Reynolds Adolescent Depression Scale [19]. The questionnaire is composed of 23 items referring to various depressive symptom categories such as mood, vegetative, cognitive, and psychomotor disturbances. The questionnaire, filled in by adolescents, is scored on a four-point scale ranging from 1 = ‘almost never’ to 4 = ‘most of the time,’ and the items scores were summed into one internalizing symptoms dimension score. Mean scores were used for the analyses. The RADS questionnaire had high internal consistency for each of the six annual waves (*α*_s_ = 0.93–0.95).

#### Adolescent externalizing symptoms

Adolescent externalizing behavior symptom dimensions were measured by the 30-item externalizing scale (that consists of the 19-item aggression behavior symptom subscale and the 11-item delinquency behavior symptom subscale) of the Youth Self-Report [20]. The questionnaire, filled in by the adolescent, is scored on a three-point scale ranging from 0 = ‘never,’ 1 = ‘sometimes,’ to 2 = ‘often’ and the items scores were summed into one externalizing symptoms dimension score. Mean scores were used for the analyses. The YSR questionnaire had high internal consistency for each of the six annual waves (*α*_s_ = 0.87–0.91).

### Strategy of Analysis

**Fig. 1:**
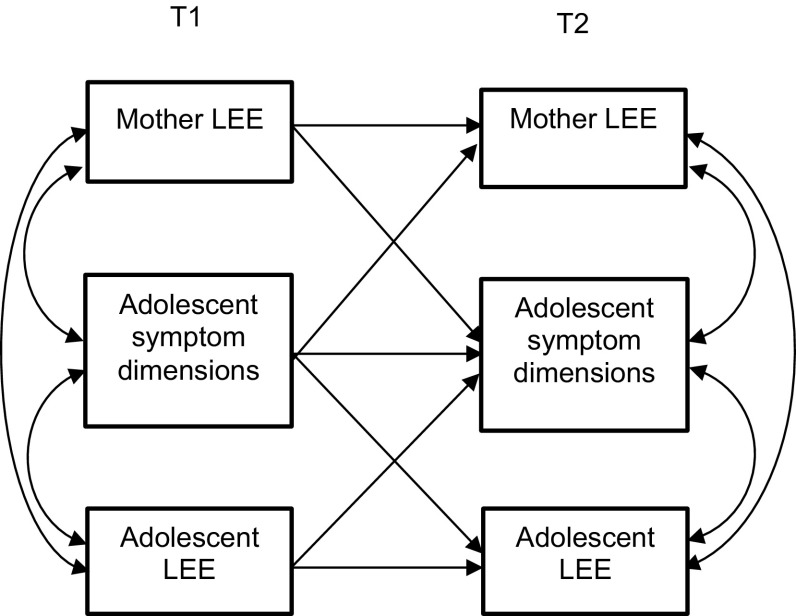
Schematization of the cross-lagged model

For unraveling reciprocal influences of maternal and adolescent perceived EE to adolescent internalizing and externalizing symptom dimensions, we tested two (i.e., one for internalizing and one for externalizing) cross-lagged panel models in Mplus. A schematization of the tested model, reported in a simplified version with two time points, is displayed in Fig. 1. In each model, we tested for cross-lagged associations between maternal and adolescent EE and problem behaviors, controlling for 1-year and 2-year stability paths and within-time correlations. Models were estimated with the robust maximum likelihood estimation method [21] to account for non-normality of internalizing and externalizing problem behaviors.

We tested the model fit by means of the (a) *χ*^2^/*df* ratio that should be lower than 3, (b) the Comparative Fit Index (CFI) that should be higher than 0.90, and (c) the Root Mean Square Error of Approximation (RMSEA) that should be lower than 0.08 [22]. To model the associations between maternal and adolescent EE and problem behaviors as parsimoniously as possible, we first tested time-invariant models, in which cross-lagged paths and T2–T6 within-time correlations were fixed to be equal across time. Thus, we compared these models with models in which cross-lagged paths were free to vary across time. If freeing cross-lagged paths did not result in an improvement in the model fit, we chose the more parsimonious (time-invariant) models as the final ones. In order to determine significant differences between models, at least two out of these three criteria had to be matched: a significant Δ*χ*^2^ test [20], ΔCFI ≥ −0.010, and ΔRMSEA ≥ 0.015 [23]. Since we tested complex models, in order to reduce probability of Type I error (i.e., rejecting the null hypothesis when the null hypothesis is true), we used the more conservative value of 0.01 as the cutoff for the level of significance.

## Results

### Descriptive statistics and correlations

Means, standard deviations, and correlations among study variables computed at the first wave are reported in Table 1 (the same statistics computed for all the other five waves can be obtained from the first author upon request). As can be seen, mother and adolescent LEE dimensions were positively and significantly correlated with adolescent internalizing and externalizing problem behaviors (only the correlation between mother intrusiveness and adolescent internalizing symptoms was not significant).

**Table 1:** Descriptive statistics and bivariate correlations among study variables computed at the first wave

	Descriptives		Correlations									
Variables	M	SD	1	2	3	4	5	6	7	8	9	10
Mother LEE												
1. Lack of emotional support	1.61	0.39	1									
2. Intrusiveness	2.29	0.48	0.12	1								
3. Irritation	2.14	0.64	0.54**	0.27**	1							
4. Criticism	1.65	0.50	0.59**	0.25**	0.55**	1						
Adolescent LEE												
5. Lack of emotional support	1.38	0.26	0.31**	0.08	0.17**	0.27**	1					
6. Intrusiveness	2.52	0.56	0.08	0.13*	0.09	0.12*	0.25**	1				
7. Irritation	1.71	0.46	0.14*	0.06	0.16**	0.16**	0.54**	0.18**	1			
8. Criticism	1.58	0.40	0.28**	0.08	0.16**	0.28**	0.68**	0.37**	0.31**	1		
Adolescent problem behaviors												
9. Internalizing	1.63	0.49	0.18**	0.01	0.13*	0.23**	0.38**	0.20**	0.16**	0.40**	1	
10. Externalizing	0.35	0.24	0.18**	0.11	0.18**	0.25**	0.40**	0.22**	0.31**	0.40**	0.47**	1

Mean scores ranged from 1 to 4 for LEE and internalizing problems, and from 0 to 2 for externalizing problems

* *p* < 0.01; ** *p* < 0.001

### Cross-lagged models

Results of model testing are reported in Table 2. As can be seen, both for the model on internalizing symptoms and for the model on externalizing symptoms, we could retain the most parsimonious solution, which is the model assuming equality of cross-lagged paths and T2–T6 within-time correlations. Ancillary multi-group analyses indicated that cross-lagged effects for both the internalizing and the externalizing models were equal across gender groups. Thus we will only discuss the results obtained in the total sample.

**Table 2:** Model fit indices and model comparisons

	Model fit indices						Model difference		
	*χ* ^2^	*df*	*χ* ^2^/*df*	CFI	TLI	RMSEA [95 % CI]	Δ*χ* ^2^ (Δ*df*)^a^	ΔCFI	ΔRMSEA
Internalizing symptoms									
1. Model with cross-lagged paths free to vary across waves	2178.31	1110	1.96	0.935	0.919	0.044 [0.041, 0.047]			
2. Model with cross-lagged paths fixed across waves	2255.31	1174	1.92	0.934	0.922	0.043 [0.040, 0.046]			
Difference between models 2 and 1							78.45(64), *p* = 0.105	−0.001	−0.001
3. Multi-group model with cross-lagged paths free to vary across gender groups	4083.49	2405	1.70	0.909	0.89	0.053 [0.050, 0.056]			
4. Multi-group model with cross-lagged paths fixed across gender groups	3809.18	2364	1.61	0.916	0.901	0.050 [0.047, 0.052]			
Difference between models 4 and 3							259.98 (41), *p* < 0.001	0.007	−0.003
Externalizing symptoms									
1. Model with cross-lagged paths free to vary across waves	2112.16	1110	1.90	0.938	0.923	0.043 [0.040, 0.045]			
2. Model with cross-lagged paths fixed across waves	2233.54	1174	1.90	0.935	0.923	0.043 [0.040, 0.045]			
Difference between models 2 and 1							121.39 (64), *p* < 0.001	−0.003	0.000
3. Multi-group model with cross-lagged paths free to vary across gender groups	4042.61	2405	1.68	0.911	0.895	0.052 [0.050, 0.055]			
4. Multi-group model with cross-lagged paths fixed across gender groups	3850.26	2364	1.63	0.914	0.899	0.050 [0.047, 0.053]			
Difference between models 4 and 3							181.90 (41), *p* < 0.001	−0.003	0.002

*χ* ^2^ Chi Square, *df* degrees of freedom, *CFI* Comparative Fit Index, *TLI* Tucker–Lewis Index, *RMSEA* Root Mean Square, *CI* Confidence Interval, *Δ* Parameter change

^a^Since the MLR estimation method was used, Δ*χ* ^2^ model comparisons are based on Satorra and Bentler’s [21] scaled difference Chi-square test statistic

### Adolescent internalizing symptoms

**Fig. 2:**
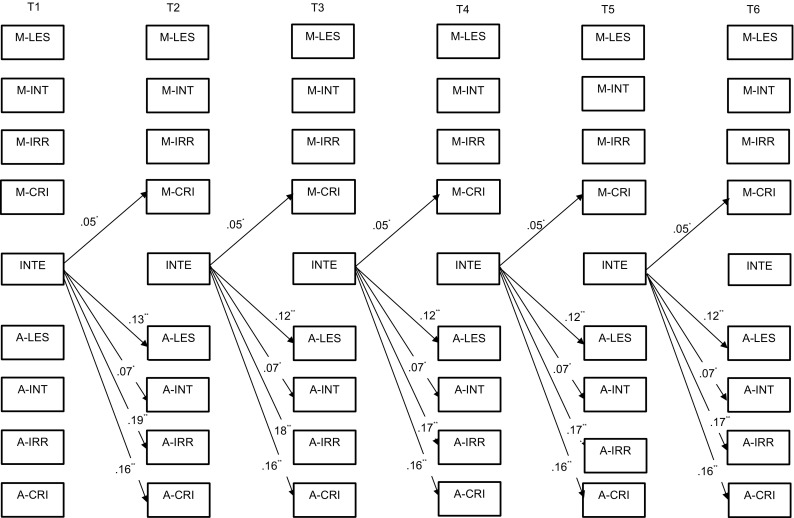
Significant standardized cross-lagged effects for the model on internalizing symptoms. *M* mother report, *A* adolescent report, *LES* lack of emotional support, *INT* intrusiveness, *IRR* irritation, *CRI* criticism, *INTE* internalizing symptoms. *p < 0.01; **p < 0.001

First, it was found that all the within-time correlations (Table 3) of the adolescents’ internalizing symptoms and the LEE dimensions were significant. Additionally, the model on reciprocal relationships between maternal and adolescent LEE and internalizing symptoms indicated a number of significant cross-lagged paths (Fig. 2). The main direction of effects was from adolescent internalizing symptoms to LEE dimensions. Specifically, internalizing symptoms predicted levels of all adolescent LEE dimensions (i.e., lack of emotional support, intrusiveness, irritation, and criticism) as well as mother criticism over the course of adolescence.

**Table 3:** Within-time correlations between maternal and adolescent LEE and internalizing and externalizing problems obtained in the cross-lagged models

	Internalizing problems		Externalizing problems	
	T1	T2–T6	T1	T2–T6
Mother LEE				
Lack of emotional support	0.18**	0.01	0.18**	0.05
Intrusiveness	0.00	−0.01	0.11	0.03
Irritation	0.13*	0.04	0.18**	0.03
Criticism	0.23**	0.05	0.25**	0.06
Adolescent LEE				
Lack of emotional support	0.38**	0.28**	0.40**	0.21**
Intrusiveness	0.20**	0.18**	0.23**	0.11*
Irritation	0.17**	0.26**	0.32**	0.19**
Criticism	0.40**	0.29**	0.40**	0.20**

Since the models with time-invariant T2–T6 correlations were retained as the final ones, we present T2–T6 correlations representing the averaged standardized coefficients over the five time intervals

* *p* < 0.01; ** *p* < 0.001

### Adolescent externalizing symptoms

**Fig. 3:**
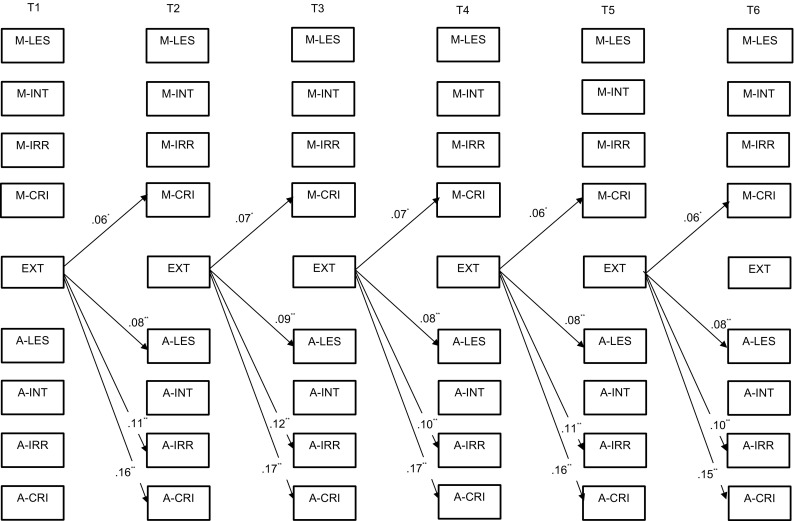
Significant standardized cross-lagged effects for the model on externalizing symptoms. *M* mother report, *A* adolescent report, *LES* lack of emotional support, *INT* intrusiveness, *IRR* irritation, *CRI* criticism, *EXT* externalizing symptoms. *p < 0.01; **p < 0.001

Here it was also found that all the within-time correlations (Table 3) of the adolescents’ externalizing symptoms and the LEE dimensions were significant. The model on reciprocal relationships between maternal and adolescent LEE and externalizing symptoms revealed that all the effects had the same direction, from adolescent externalizing symptoms to LEE dimensions (Fig. 3). More specifically, adolescent externalizing symptoms predicted three out of four adolescent LEE dimensions (i.e., lack of emotional support, irritation, and criticism) and mother criticism.

## Discussion

This 6-year, longitudinal study found that both internalizing and externalizing symptoms predicted the adolescent’s perception of maternal expressed emotion over time. Furthermore, both internalizing and externalizing symptom dimensions predicted the mother’s perception of her own EE criticism toward her adolescent over time. It should be noted that this is the first longitudinal study that included both the adolescent’s and the mother’s perceptions of the different facets of the EE household environment when studying the effects EE and adolescent psychopathological symptoms have on one another. In a nutshell, this study demonstrated that adolescent psychopathological symptom dimensions are predictive of an adolescent’s perceptions of the EE household environment and the mother’s perception of her own EE criticism.

Presently, in EE theory, it is commonly held that high EE household environments help enhance adolescent psychopathological distress (an EE effect model). This view is based on Hooley’s central hypothesis [24, 25] that high EE in relatives (such as mothers) reflects their underlying beliefs that the patient (such as adolescents) could do more to control their psychopathological symptoms if the adolescent desired to do so and that the failure to control their psychopathological symptoms is due to a unique intrapersonal factor of the patient (for example, a personal habit). This is also known in psychotherapies that focus on high EE household environments as “blaming the patient.” According to a highly cited review of the literature on relative EE and patient psychopathological symptoms, the authors state that all of the published investigations in their literature review of adults and children confirmed this hypothesis [26]. However, the authors do note that a limitation is that most of the studies that they included in their review are cross-sectional and they only employ correlational analyses.

Importantly, in this 6-year longitudinal study of maternal EE and adolescent psychopathological symptoms, it was demonstrated that a psychopathological effect model (the adolescent’s psychopathological symptoms) is a better explanation of the association between parent/adolescent EE and adolescent psychopathological symptom dimensions (in other words, adolescent symptoms enhancing EE) than an EE effect model. These findings seem to contradict the commonly held view that high EE household environments help enhance adolescent psychopathological distress.

This point leads back to considerations about family treatments of adolescent internalizing and externalizing symptom dimensions. Most family treatments that employ the EE concept focus on an EE effects model [the EE provider (i.e., the mother) affecting the EE receiver (i.e., the child)] [27]. A psychopathological effects model, in which the child’s psychopathological symptoms elicit EE from the mother, has received much less attention in therapies designed to reduce EE. It is quite conceivable that both a psychopathological effects model (as has been found in this study) as well as an EE effect model (as has been found in previous studies such as Hale et al. [9, 18]) help explain the relationship between maternal EE and the course of adolescent internalizing and externalizing symptoms. Specifically, in Cognitive Therapy, a major focus of the therapy is on the beliefs a person holds as to his or her interactions with others. This “belief” is literally the person’s perception of the interactions he or she has with others. Hence it is possible that psychotherapies that use the EE concept could be refined to incorporate these divergent perceptions on the part of the mother as well as the part of the adolescent.

With respect to the limitations of this study, it should be noted that EE was only measured with the LEE and that the CFI interview was not used. In an overview of the measures of EE, it has been discussed whether questionnaire-based measures of EE measure EE in the same way as the CFI interview does, while also raising the point that clinically useful and accessible EE alternatives for the CFI interview are needed [28]. Therefore, it is not possible to judge if the LEE findings of this study would be similar to the CFI interview measured EE and future studies are recommended to address this issue. However, as previously stated, the LEE has good psychometric properties and an advantage that the LEE holds over the CFI is that the LEE can be quickly administrated and scored, as opposed to the CFI (which takes several hours to conduct and code) thereby better allowing for prospective, longitudinal studies addressing effects of EE.

Secondly, with respect to the LEE, the results of this study are limited by only studying maternal EE and not collecting paternal EE data as well. We would suggest that future research should also collect the fathers’ EE scores since previous research has shown that there is a unique interplay between an adolescent’s internalizing symptoms and his/her father’s behaviors which is not necessarily the same as the mother’s behaviors [29].

Finally, it should also be noted that the correlations between the mothers’ and the adolescents’ rated LEE scores were significant, but low (see Table 1). As noted by Bögels and Van Melic [30], low correlations between child and parent ratings of parental behaviors commonly occur in such studies and they suggest that a reason for this occurrence might be personal biases associated with the child’s perspective and the mother’s own perspective. While direct observation of behaviors might help solve this perspective problem, these same authors also suggest that an advantage that questionnaires have over home observations is that questionnaires are less intrusive and the respondent’s “answers are based on home observations and infinite samples of behavior across infinite situations and tasks” (p. 1585) [30].

Additionally, this study focused only on self-reports of internalizing and externalizing symptoms from adolescents from the general community. This should not be confused with a clinical diagnosis of a psychiatric disorder. A structured clinical interview could have been used to help determine the strength of the relationship between the adolescents’ self-reports of internalizing and externalizing symptoms and an actual diagnosis of these related disorders. Moreover, these adolescents came from the general community, whereas many previous studies of EE and adolescent internalizing and externalizing symptoms came from clinical populations. However, it has also been suggested that prospective longitudinal community studies of psychopathological symptom dimensions may help circumvent the problem of referral bias that frequently occurs in the clinical setting and may better characterize the course of developmental psychopathological symptoms [31]. Nevertheless, future studies in the clinical setting should be conducted to replicate these findings.

It could also be asked if self-reports by 13 year olds (the first wave of this study) could be considered accurate. The questionnaires that were used for the internalizing (RADS) and externalizing (YSR) symptom dimensions have shown good psychometric properties in various studies for children of 13 years of age or younger (RADS: e.g., [32]; YSR: e.g., [33]). However, there has been much less study of age groups with EE questionnaires such as the LEE. Previous studies have found the psychometric properties of the LEE with adolescents of 13 years or older to be good [9, 18]. Still, there have been much less studies with the LEE with adolescents as there has been with the RADS and the YSR. Hence, future studies with the LEE in adolescent populations may further address this current issue.

In conclusion, the results of this longitudinal EE study that followed adolescents from 13 to 18 years of age found that both internalizing and externalizing symptom dimensions predicted the adolescent’s perception of maternal EE as well as the mother’s own rated EE criticism over time. As was previously noted, in EE theory, it is commonly held that high EE household environments help enhance adolescent psychopathological distress (an EE effect model). However, this study found a psychopathological effect model contradicting the commonly held view that high EE household environments help enhance adolescent psychopathological distress. The findings of this study should give both researchers and therapists a reason to reevaluate only using the EE effects model assumption in future EE studies.

## References

[1] Brown GW, Leff JP, Vaughn CE (1985). The discovery of expressed emotion: induction or deduction?. Expressed emotion in families: its significance for mental illness.

[2] Leff J, Vaughn C (1985). Expressed emotion in families: its significance for mental illness.

[3] Asarnow JR, Tompson M, Woo S, Cantwell D (2001). Is expressed emotion a specific risk factor for depression or a nonspecific correlate of psychopathology?. J Abnorm Child Psychol.

[4] Hirshfeld DR, Biederman J, Brody L, Faraone SV, Rosenbaum JF (1997). Associations between expressed emotion and child behavioral inhibition and psychopathology: a pilot study. J Am Acad Child Adolesc Psychiatry.

[5] Peris TS, Hinshaw SP (2003). Family dynamics and preadolescent girls with ADHD: the relationship between expressed emotion, ADHD symptomatology, and comorbid disruptive behavior. J Child Psychol Psychiatry.

[6] Psychogiou L, Daley DM, Thompson MJ, Sonuga-Barke EJS (2007). Mothers’ expressed emotion toward their school-aged sons: associations with child and maternal symptoms of psychopathology. Eur Child Adolesc Psychiatry.

[7] Hooley JM, Teasdale JD (1989). Predictors of relapse in unipolar depressives: expressed emotion, marital distress, and perceived criticism. J Abnorm Psychol.

[8] Hale WW, Van der Valk I, Akse J, Meeus WHJ (2008). The interplay of early adolescent depressed mood, aggressive behavior and perceived parental rejection: a four year longitudinal community study. J Youth Adolesc.

[9] Hale WW, Raaijmakers QAW, Van Hoof A, Meeus WHJ (2011). The predictive capacity of perceived expressed emotion as a dynamic entity of adolescents from the general community. Soc Psychiatry Psychiatr Epidemiol.

[10] Nelemans SA, Hale WW, Branje SJT, Hawk ST, Meeus WHJ (2014). Maternal criticism and adolescent depressive and generalized anxiety disorder symptoms: a 6-year longitudinal community study. J Abnorm Child Psychol.

[11] Cole JD, Kazarian SS (1988). The level of expressed emotion scale: a new measure of expressed emotion. J Clin Psychol.

[12] Gerlsma C, Van der Lubbe PM, Van Nieuwenhuizen C (1992). Factor analysis of the level of expressed emotion scale, a questionnaire intended to measure “perceived expressed emotion”. Br J Psychiatry.

[13] Gerlsma C, Hale WW (1997). Predictive power and construct validity of the level of expressed emotion (LEE) scale: depressed out-patients and couples from the general community. Br J Psychiatry.

[14] Hale WW, Keijsers L, Klimstra TA, Raaijmakers QAW, Hawk S, Branje SJT, Frijns T, Wijsbroek SAM, Van Lier P, Meeus WHJ (2011). How does longitudinally measured maternal expressed emotion affect internalizing and externalizing symptoms of adolescents from the general community?. J Child Psychol Psychiatry.

[15] Little RJA (1988). A test of missing completely at random for multivariate data with missing values. J Am Stat Assoc.

[16] Muthén LK, Muthén BO (2012). Mplus user’s guide.

[17] Enders CK, Bandalos DL (2001). The relative performance of full information maximum likelihood estimation for missing data in structural equation models. Struct Equ Modeling.

[18] Hale WW, Raaijmakers QAW, Gerlsma J, Meeus WHJ (2007). Does the level of expressed emotion (LEE) questionnaire have the same factor structure for adolescents as it has for adults?. Soc Psychiatry Psychiatr Epidemiol.

[19] Reynolds WM (2000). Reynolds Adolescent Depression Scale (RADS-2) professional manual.

[20] Verhulst FC, Van der Ende J, Koot HM (1997). Handleiding voor de Youth Self-Report (YSR) [manual for the Youth Self-Report].

[21] Satorra A, Bentler PM (2001). A scaled difference Chi square test statistic for moment structure analysis. Psychometrika.

[22] Kline RB (2011). Principles and practice of structural equation modeling.

[23] Chen FF (2007). Sensitivity of goodness of fit indexes to lack of measurement invariance. Struct Equ Modeling.

[24] Hooley JM (1985). Expressed emotion: a review of the critical literature. Clin Psychol Rev.

[25] Hooley JM, Miklowitz DJ, Beach SRH, Beach SRH, Wamboldt MZ, Kaslow NJ, Heyman RE, First MB (2006). Expressed emotion and DSM-V. Relational processes and DSM-V: neuroscience, prevention and treatment.

[26] Barrowclough C, Hooley JM (2003). Attribution and expressed emotion: a review. Clin Psychol Rev.

[27] Hooley JM, Hahlweg K, Goldstein MJ (1987). The nature and origins of expressed emotion. Understanding major mental disorder: the contribution of family interaction research.

[28] Hooley JM, Parker HA (2006). Measuring expressed emotion: an evaluation of the shortcuts. J Fam Psychol.

[29] Bögels SM, Phares V (2008). Fathers’ role in the etiology, prevention and treatment of child anxiety: a review and new model. Clin Psychol Rev.

[30] Bögels SM, Van Melick M (2004). The relationship between child-report, parent self-report, and partner report of perceived parental rearing behaviors and anxiety in children and parents. Pers Individ Differ.

[31] Hale WW, Raaijmakers QAW, Muris P, Van Hoof A, Meeus WHJ (2009). One factor or two parallel processes? Comorbidity and development of adolescent anxiety and depressive disorder symptoms. J Child Psychol Psychiatry.

[32] Reynolds WM, Mazza JJ (1998). Reliability and validity of the Reynolds Adolescent Depression Scale with young adolescents. J Sch Psychol.

[33] Ivanova MY, Achenbach TM, Rescorla LA, Dumenci L, Almqvist F, Bilenberg N, Bird H, Broberg AG, Dobrean A, Döpfner M, Erol N, Forns M, Hannesdottir H, Kanbayashi Y, Lambert MC, Leung P, Minaei A, Mulatu MS, Novik T, Oh KJ, Roussos A, Sawyer M, Simsek Z, Steinhausen HC, Weintraub S, Winkler Metzke C, Wolanczyk T, Zilber N, Zukauskiene R, Verhulst FC (2007). The generalizability of the Youth Self-Report syndrome structure in 23 societies. J Consult Clin Psychol.

